# Microfluidic Impedance Flow Cytometry Enabling High-Throughput Single-Cell Electrical Property Characterization

**DOI:** 10.3390/ijms16059804

**Published:** 2015-04-29

**Authors:** Jian Chen, Chengcheng Xue, Yang Zhao, Deyong Chen, Min-Hsien Wu, Junbo Wang

**Affiliations:** 1 State Key Laboratory of Transducer Technology, Institute of Electronics, Chinese Academy of Sciences, Beijing 100190, China; E-Mails: chenjian@mail.ie.ac.cn (J.C.); xuechengcheng13@mails.ucas.ac.cn (C.X.); zhaoyang110@mails.ucas.ac.cn (Y.Z.); dychen@mail.ie.ac.cn (D.C.); 2 Graduate Institute of Biochemical and Biomedical Engineering, Chang Gung University, Taoyuan 333, Taiwan

**Keywords:** microfluidics, single-cell electrical property analysis, impedance flow cytometry, high throughput

## Abstract

This article reviews recent developments in microfluidic impedance flow cytometry for high-throughput electrical property characterization of single cells. Four major perspectives of microfluidic impedance flow cytometry for single-cell characterization are included in this review: (1) early developments of microfluidic impedance flow cytometry for single-cell electrical property characterization; (2) microfluidic impedance flow cytometry with enhanced sensitivity; (3) microfluidic impedance and optical flow cytometry for single-cell analysis and (4) integrated point of care system based on microfluidic impedance flow cytometry. We examine the advantages and limitations of each technique and discuss future research opportunities from the perspectives of both technical innovation and clinical applications.

## 1. Introduction

Single-cell electrical properties (e.g., membrane capacitance or cytoplasm resistance) can be utilized as cellular biophysical markers to evaluate cellular status in a label-free manner [1,2]. They have been demonstrated to classify various types of tumor cells [3,4,5], stem cells [6] and blood cells [7,8,9,10,11].

Conventionally there are mainly three techniques capable of characterizing single-cell electrical properties: dielectrophoresis, patch clamping and electrorotation [12]. Dielectrophoresis is demonstrated to quantify cellular electrical properties by curve fitting of the Clausius–Mossotti factor spectra or cell count spectra. However, since the spectra are not from the measurements of the same cells, only average electrical properties of a cell population can be obtained [3,4,12,13,14,15,16,17]. Patch-clamp devices characterize the activities of cellular ion channels by sucking a portion of cell membrane into a micropipette tip to form a high electrical resistance seal, enabling the quantification of specific membrane capacitance of single cells (an intrinsic size-independent electrical parameter of cells) [18,19,20,21,22,23,24]. In electro-rotation, a rotating electric field is exerted to rotate a suspended single cell as a result of Maxwell-Wagner polarization. By measuring the rotating rate as a function of the applied frequency, this method is capable of collecting membrane permittivity and cytoplasm conductivity of single cells [25,26,27,28,29,30,31,32]. However, patch clamping and electrorotation rely on the precise manipulation and positioning of pipettes (patch clamping) or cells (electrorotation) which is time-consuming and labor-intensive [12,33,34,35,36]. This could greatly affect the measurement efficiency and therefore hamper the wide application of using these techniques to acquire statistically-meaningful data.

Microfluidics is the science and technology on the processing and manipulation of small amounts of fluids (10^−9^ to 10^−18^ liters) in channels with dimensions of tens of micrometers [37,38,39]. The micrometer dimension well matches with the size of typical biological calls, making microfluidics an ideal platform for cell studies [40,41,42,43,44]. Based on the advantageous features of microfluidic technologies, microfluidics has been used for characterizing the biochemical (e.g., gene and protein) and/or biophysical properties (mechanical and electrical) of cells at the single-cell level [45,46,47,48,49,50,51].

Microfluidics-based devices for the characterization of single-cell electrical properties have been proposed, in which two major approaches, the micro electrical impedance spectroscopy (μ-EIS) and microfluidic impedance flow cytometry [12,35,36], are commonly used. μ-EIS is a non-invasive approach to characterize immobilized single cells between two electrodes relying on hydrodynamic fluid trapping [52,53,54,55,56,57,58], vacuum aspiration [59,60,61,62,63,64,65], dielectrophoretic forces [66,67,68,69] or surface modifications [70,71,72]. Although this technique can conduct spectroscopy sweeping on the trapped single cells, it normally suffers from limited throughput and thus might not be suitable for collecting data from large amounts of cells [12,33,34,35,36].

Meanwhile, microfluidic impedance flow cytometry has also been demonstrated where single cells are pushed to continuously flow through two microelectrodes in which the impedance data of cells at multiple frequencies are measured [35,36]. Compared to the conventional coulter counters which rely on DC or low-frequency signal for cell size characterization [73,74,75,76], the multiple-frequency-based impedance data obtained from the microfluidic impedance flow cytometry enable the characterization of cellular sizes, membrane capacitance and cytoplasm resistance in a high-throughput manner [35,36].

In this review, we focus on the recent advances of the four perspectives of microfluidics-based flow cytometers for single-cell electrical property characterization: (1) early developments of microfluidic impedance flow cytometry for single-cell electrical property characterization; (2) microfluidic impedance flow cytometry with enhanced sensitivity; (3) microfluidic impedance and optical flow cytometry for single-cell analysis and (4) integrated point of care system based on microfluidic impedance flow cytometry (see Table 1).

**Table 1:** Key developments in the field of microfluidic impedance flow cytometry enabling high-throughput cellular electrical property characterization.

Techniques	Quantified Parameters	Classified Objects and Key Observations	References
Coplanar microelectrodes	Two-frequency impedance data (1.7 and 15.0 MHz)	Polymer beads of 5 and 8 μm, normal erythrocytes and their ghost counterparts	[ 77]
Coplanar microelectrodes	One-frequency impedance data (100 kHz)	Liver tumor cells at normal, apoptotic and necrotic status, leukemia cells	[ 78]
Coplanar microelectrodes	One-frequency impedance data (2.0 MHz)	Different stages of *P. falciparum* infected red blood cells and uninfected red blood cells	[ 9]
Parallel microelectrodes	Two-frequency impedance opacity |Z_high_|/|Z_ref_| (f_ref_ = 602 kHz, f_high_ = 350 kHz–20.0 MHz )	Polymer beads of 5, 6 μm, red blood cells and their fixed counterparts	[ 79]
Parallel microelectrodes	Two-frequency impedance opacity |Z_high_|/|Z_ref_| (f_ref_ = 500 kHz, f_high_ = 0.5–250.0 MHz)	Wild-type yeasts and a mutant with different sizes and distribution of vacuoles in the intracellular fluid	[ 80]
Parallel microelectrodes + insulating fluid focusing	One-frequency impedance data (503 kHz)	Polymer beads of 1, 2 μm, and *E coli*	[ 81]
Parallel microelectrodes + resonance	Two-frequency impedance data (87.2 and 89.2 MHz)	*E. coli*, *B. subtilis* and polymer beads of 2 μm	[ 82]
Constriction channel	One-frequency impedance data (100 kHz)	Size-comparable tumor cells and their more malignant counterparts	[ 83]
Constriction channel	One-frequency impedance data (100 kHz)	Adult red blood cells and neonatal red blood cells	[ 84]
Constriction channel	Four-frequency impedance data (50 kHz, 250 kHz, 500 kHz and 1.0 MHz)	Polymer beads of 20 μm, undifferentiated stem cells and differentiated stem cells	[ 6]
Constriction channel + equivalent circuit model	Specific membrane capacitance and cytoplasm conductivity	Characterization of size-independent intrinsic cellular electrical properties from hundreds of single cells	[ 85]
Constriction channel + equivalent circuit model	Specific membrane capacitance and cytoplasm conductivity	Paired high- and low-metastatic cancer cells, and tumor cells with single oncogenes under regulation	[ 5]
Parallel microelectrodes + optical lens	Two-frequency impedance data (503 kHz and 1.7 MHz) and fluorescent signals	lymphocytes, monocytes and neutrophils	[ 10]
Parallel microelectrodes + optical lens	Two-frequency impedance data (503 kHz and 10.0 MHz) and fluorescent signals	Lymphocytes, lymphocytes + CD4 beads, granulocytes, monocytes and monocytes + CD4	[ 11]
Parallel microelectrodes + on-chip optical fibers	One-frequency impedance data (1.0 MHz), fluorescent signals, and side scattered light	Microbeads (10 and 15 μm diameter fluorescent, 20 and 25 μm diameter plain)	[ 86]
Parallel microelectrodes + on-chip waveguides	Two-frequency impedance data (500 kHz and 2.0 MHz), fluorescent signals, and side scattered light	Lymphocytes, granulocytes, monocytes, neutrophils and CD4 labelled white blood cells	[ 87]
Parallel microelectrodes + sample pretreatment module	Two-frequency impedance data (500 kHz and 1.7 MHz)	Lymphocytes, monocytes, neutrophils, red blood cells and platelets	[ 88]
Parallel microelectrodes + sample pretreatment module	Two-frequency impedance data (303 kHz and 1.7 MHz)	CD4^+^ and CD8^+^ lymphocytes	[7]

## 2. Early Development of Microfluidic Flow Cytometry for Single-Cell Electrical Property Characterization

**Figure 1:**
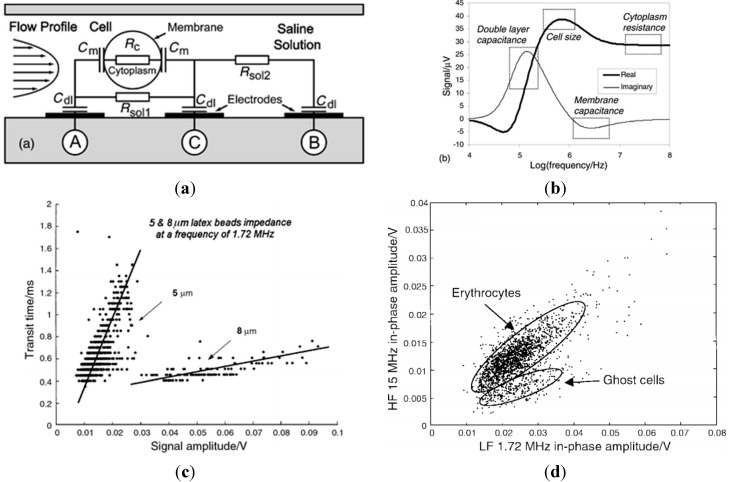
(**a**) The first-generation microfluidic impedance flow cytometry where a microfluidic chip with integrated channels and a differential pair of coplanar microelectrodes were proposed to quantify two-frequency impedance data of single cells flushed through the measurement area in a high-throughput manner; (**b**) The complex impedance spectrum of a cell is simulated using an equivalent circuit model where impedance data at various frequency domains indicate the electrical double layer, cellular size, membrane capacitance and cytoplasm resistance, respectively; (**c**) Impedance amplitude difference of 5 and 8 μm latex beads, confirming that impedance data at ~1 MHz can reflect particle sizes. Note that “transit time” indicates the traveling velocity of latex beads which were also obtained from impedance data; (**d**) Normal erythrocytes and erythrocyte ghost cells were characterized, with comparable low-frequency impedance data indicating size comparability and significant differences at high-frequency impedance data suggesting cytoplasm conductivity differences [77].

Renaud *et al.* are the pioneers in the field of microfluidic impedance flow cytometry [77,79,89,90,91,92,93]. In 2001, Renaud *et al.* proposed the first microfluidics-based impedance flow cytometry for high-throughput single-cell electrical property characterization [77]. As shown in Figure 1a, a microfluidic chip with channels integrated with a differential pair of coplanar microelectrodes was used to characterize electrical properties of single cells. The cells were flushed through the measurement area in a high-throughput manner with the impedance data measured at two given frequencies. In this study, an equivalent circuit model for microfluidic impedance flow cytometry was developed where C_m_, R_c_, R_sol_ and C_dl_ represent cell membrane capacitance, cytoplasm resistance, buffer solution resistance and electrical double layer capacitance, respectively (see Figure 1a).

In addition, complex impedance spectrum of a cell as simulated using an equivalent circuit model was shown in Figure 1b. Based on simulation results, the authors suggested that the impedance data for frequencies lower than 100 kHz, between 100 kHz–1 MHz, 2–5 MHz and 10–100 MHz reflect the electrical double layer, cellular size, membrane capacitance and cytoplasm resistance, respectively. Note that this impedance spectrum has served as the guiding rule of frequency choice in the subsequent development of microfluidic impedance flow cytometry.

To demonstrate its applications, the microfluidic device was used to differentiate latex beads of 5 and 8 μm at 1.72 MHz. The result confirmed that impedance data at ~1 MHz does reflect particle sizes (see Figure 1c). Furthermore, normal erythrocytes and erythrocyte ghost cells (namely the erythrocytes with cytoplasm replaced with phosphate buffer solution) were characterized and differentiated. The impedance data for these two types of cells were found similar at 1.72 MHz indicating comparable cell sizes whereas, significantly different at 15 MHz suggesting differences in cytoplasm conductivity (see Figure 1d).

**Figure 2:**
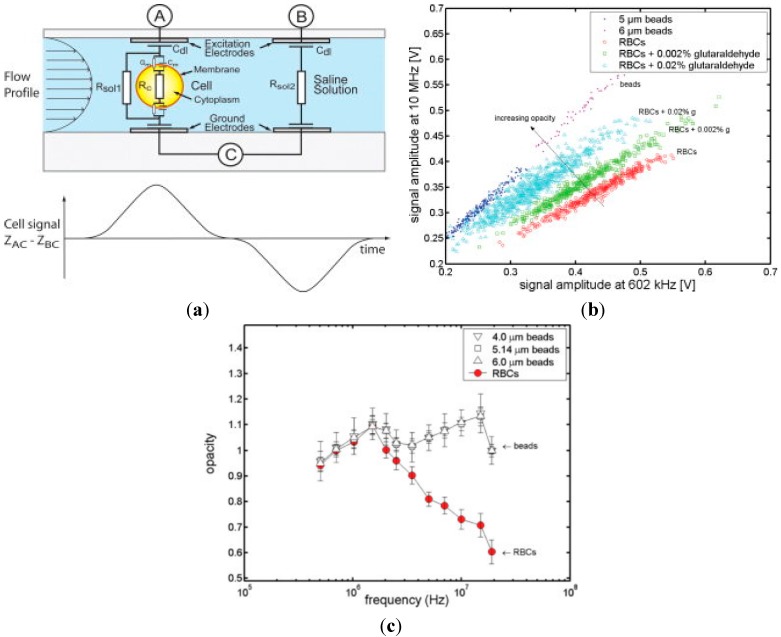
(**a**) The second-generation microfluidic impedance flow cytometry where the parallel overlap micro electrodes were used to replace the previously reported coplanar micro electrodes; (**b**) Two-frequency impedance data of polystyrene beads, normal red blood cells and fixed red blood cells, which can be classified to an extent based on opacity defined as |Z_high_|/|Z_ref_|; (**c**) Opacity spectrum of red blood cells and polystyrene beads where no significant difference was noticed among the opacity spectra for polystyrene beads of different diameters, confirming that opacity can be used to normalize the particle size. In addition, a decrease in opacity at the high frequency domain of red blood cells compared to polystyrene beads was observed, confirming that the cytoplasm of red blood cells is more conductive than polystyrene beads [79].

In 2005, Renaud *et al.* proposed the second-generation microfluidic impedance flow cytometry [79] where the parallel overlap microelectrodes were used to replace the previously reported coplanar microelectrode, enabling the production of more homogeneous current density around the single cells under measurement (see Figure 2a). Furthermore, systematic experiments were conducted to classify polystyrene beads (5 and 6 μm), normal red blood cells and fixed red blood cells based on the impedance data at the frequency of 602 kHz and 10 MHz (see Figure 2b).

In this study, opacity was defined as |Z_high_|/|Z_ref_| to partially remove the dependence of the impedance data on particle sizes. As shown Figure 2c, no significant difference was noticed among the opacity spectrum (f_ref_ = 602 kHz)) for polystyrene beads of 4.0, 5.1, and 6.0 μm diameters, confirming that, to an extent, opacity is insensitive to particle sizes. In addition, a decrease in opacity at the high frequency domain of red blood cells compared to polystyrene beads was observed, confirming that the cytoplasm of red blood cells is more conductive than polystyrene beads. As a valuable impedance parameter, opacity has been commonly used in the subsequent development of microfluidic impedance flow cytometry to evaluate electrical properties of single cells.

## 3. Microfluidic Impedance Flow Cytometry with Enhanced Sensitivity

The drawback of the microfluidic impedance flow cytometry reported by Renaud *et al.* is the lack of close contact between cells and electrodes when the cells were continuously flushed to flow through the detection area between two electrodes. This issue could lead to current leakage where electric signals circumvent the cells under measurement by travelling through solutions surrounding the cells. In addition, the relative positions of travelling single cells between two facing electrodes (*i.e.*, in the middle of two facing electrodes *vs.* alongside the boundary of one detecting electrode) can also lead to issues of low detection stability and repeatability. In order to address these issues, the detection area of the microfluidic impedance flow cytometry needs to be further reduced. Two approaches have been developed to this end: sandwiching cells between two insulating fluid layers (e.g., insulating fluid flow [81,94]) or confining cells within solid constriction channels (cross sectional area smaller than biological cells) [63,83,84].

**Figure 3:**
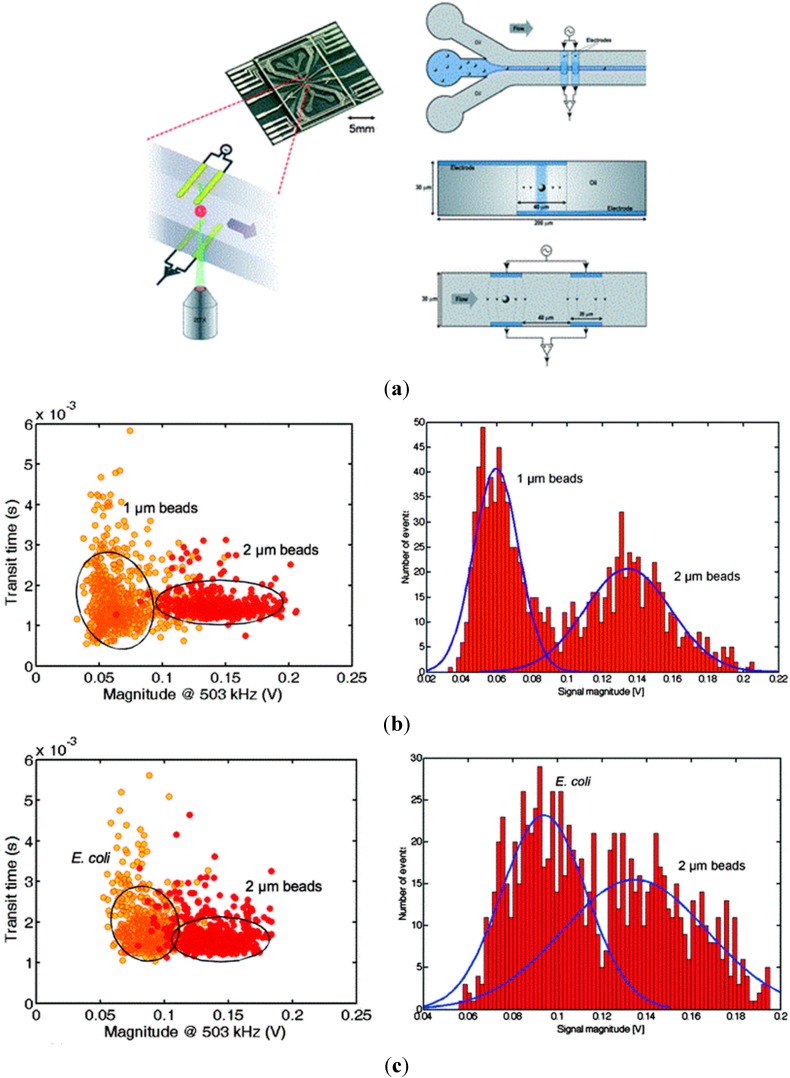
(**a**) A microfluidic impedance flow cytometer uses an insulating fluid to hydrodynamically focus a sample stream of cells suspended in electrolyte through the sensing area of two microelectrodes; (**b**) Successful classification of 1 and 2 μm diameter polystyrene beads based on impedance amplitudes at 503 kHz; (**c**) As to the classification of 2 μm diameter polystyrene beads and *E coli* (~2 μm in length and 0.5 μm in width), a significant overlap in the impedance amplitude histogram at 503 kHz was observed [81].

As the first demonstration, Morgan *et al.* developed a microfluidic impedance flow cytometer which utilized an insulating fluid to hydrodynamically focus a sample stream of cells suspended in electrolyte through the sensing area between two microelectrodes [81] (see Figure 3a). The focusing technique enhanced the measurement sensitivity without reducing the dimensions of the microfluidic channels so that channel blockage can be avoided. This microfluidic platform was used to successfully classify polystyrene beads with diameters of 1 and 2 μm based on impedance amplitudes at the frequency of 503 kHz (see Figure 3b). As to the classification of 2 μm diameter polystyrene beads and *E. coli* (~2 μm in length and 0.5 μm in width), a significant overlap in the impedance amplitude histogram was observed, which may result from the comparable sizes between 2 μm beads and *E. coli* (see Figure 3b). In this study, only one frequency at 503 kHz was used, which was previously demonstrated as the frequency enabling particle size quantification [77,79]. More frequencies higher than 503 kHz may be further used to characterize the electrical properties of *E. coli*.

Although this technique can, to an extent, address the current leakage problem by sandwiching the detection solution between two insulating fluid flows, this type of sandwiching is only one dimensional and produces a vertical conductive sheet of cells (see Figure 3a, top view and side view), which still suffers from the current leakage. In addition, the impedance data of cells can also be affected by the z-direction position of single cells during the measurement [95], which leads to additional concerns on the measurement accuracy in this microfluidic system.

To tackle the technical hurdle, a constriction channel design was put forward to further decrease the current leakage and enable single-cell electrical property characterization [63,65,83,84]. The constriction channel design with a cross-section area smaller than that of biological cells was initially used for the characterization of cellular mechanical properties (e.g., red blood cells [84,96,97,98], white blood cells [99] and tumor cells [83,100,101,102]), by which single cells were aspirated into the constriction channel with their entry times adopted as a biophysical marker.

**Figure 4:**
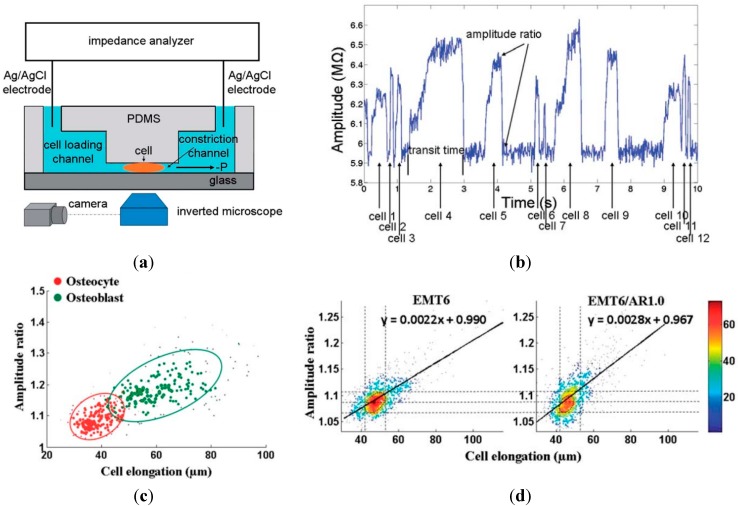
(**a**) The constriction channel based microfluidic impedance flow cytometry where single cells were aspirated through a constriction continuously while cell elongations and single frequency impedance profiles are measured simultaneously; (**b**) Raw impedance data of single cells, recording higher impedance amplitudes during cellular squeezing through the constriction channel; (**c**) The scatter plot of impedance amplitude ratio *vs.* cell elongation length for osteocytes and osteoblasts. Compared with osteocytes, osteoblasts have a larger cell elongation length and a higher impedance amplitude ratio; (**d**) The scatter plot of impedance amplitude ratio *vs.* cell elongation length for tumor cell EMT6 and their more malignant counterparts EMT6/AR 1.0, revealing a linear trend between cell elongation length and impedance amplitude ratio with different slopes and different *y*-axis intersections Reproduced by permission of the Royal Scoeity of Chemistry [83].

In 2011, Sun *et al.* proposed the first microfluidic impedance flow cytometry based on the constriction channel design, where single cells were continuously aspirated through the constriction channel while cell elongations and single-frequency impedance profiles were measured simultaneously (see Figure 4a) [83]. When a cell is aspirated through the constriction channel, it blocks electric fields and leads to a higher impedance amplitude value, which is used as an indicator of cellular electrical properties (see Figure 4b). This technique was used to classify two types of bone cells (osteoblasts *vs.* osteocytes) using a constriction channel of 6 μm × 6 μm in dimensions (at 100 kHz). To quantify the overall impedance of the cell, an impedance amplitude ratio was adopted which is defined as the ratio between the highest impedance amplitude value captured while cells are squeezed through the constriction channel and the background impedance amplitude value without cells. Compared with osteocytes, osteoblasts were found to have a larger cell elongation length and a higher impedance amplitude ratio (see Figure 4c).

The constriction channel design (8 μm × 8 μm at 100 kHz) was then used to characterize tumor cells EMT6 and their more malignant counterparts EMT6/AR 1.0, revealing a linear trend between the cell elongation length and the impedance amplitude ratio with different slopes and different *y*-axis intersections (see Figure 4d).

**Figure 5:**
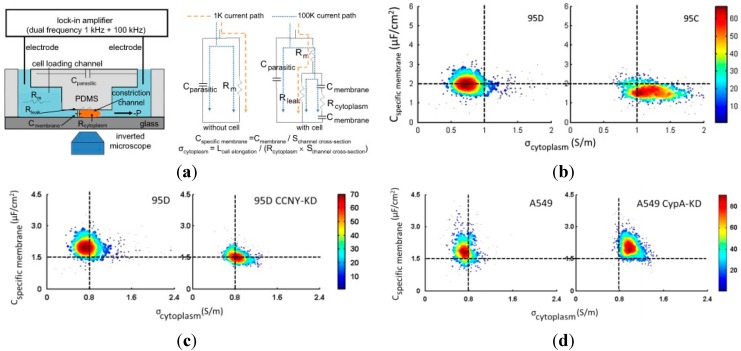
(**a**) The microfluidic impedance flow cytometry for continuous characterization of specific membrane capacitance (C_specific membrane_) and cytoplasm conductivity (σ_cytoplasm_) of single cells. Cells are aspirated continuously through the constriction channel with impedance data at 1 and 100 kHz measured simultaneously where 1 kHz impedance data were used to evaluate cellular sealing properties with constriction channel walls while 100 kHz impedance data were used to quantify C_specific membrane_ and σ_cytoplasm_ [85]; (**b**) For paired high- and low-metastatic carcinoma strains 95D and 95C cells, significant differences in both C_specific membrane_ and σ_cytoplasm_ were observed; (**c**) A statistically significant difference only in C_specific membrane_ was observed for 95D cells and 95D CCNY-KD cells with single oncogene *CCNY* down regulation (CCNY is a membrane-associated protein); (**d**) A statistically significant difference only in σ_cytoplasm_ was observed for A549 cells and A549 CypA-KD cells with single oncogene *CypA* down regulatio n (CypA is a cytosolic protein) [5].

Furthermore, based on equivalent circuit models and two-frequency measurements, these raw impedance data were translated to intrinsic cellular electrical parameters including specific membrane capacitance (C_specific membrane_) and cytoplasm conductivity (σ_cytoplasm_) [5,85,103,104]. As shown in Figure 5a, when a cell is squeezed into the constriction channel, there is an increase in amplitude and a decrease in phase for the impedance data at the frequency of 1 and 100 kHz. The 1 kHz impedance data was used to evaluate cellular sealing properties with constriction channel walls to obtain R_leak_ while 100 kHz impedance data was used to quantify C_membrane_ and R_cytoplasm_, which were then translated to C_specific membrane_ and σ_cytoplasm_ (see Figure 5a) [85]. Based on the above translations, the C_specific membrane_ and σ_cytoplasm_ of tumor cells with different types were quantified [5].

For paired high- and low-metastatic carcinoma strains 95D and 95C cells, significant differences in both C_specific membrane_ and σ_cytoplasm_ were observed (see Figure 5b). In addition, a statistically significant difference only in C_specific membrane_ was observed for 95D cells and 95D CCNY-KD cells with single oncogene CCNY down regulation (CCNY is a membrane-associated protein) (see Figure 5c). Furthermore, a statistically significant difference only in σ_cytoplasm_ was observed for A549 cells and A549 CypA-KD cells with single oncogene CypA down regulation (CypA is a cytosolic protein) (see Figure 5d).

Although the combination of impedance flow cytometry with the constriction channel design can adequately tackle the current leakage issue, the use of constriction channel could reduce the detection throughput and may lead to channel blockage. Thus, the detection throughput in the constriction channel-based microfluidic impedance flow cytometry is normally lower than its conventional counterparts [77,79,81].

## 4. Microfluidic Impedance and Fluorescent Flow Cytometry for Single-Cell Analysis

**Figure 6:**
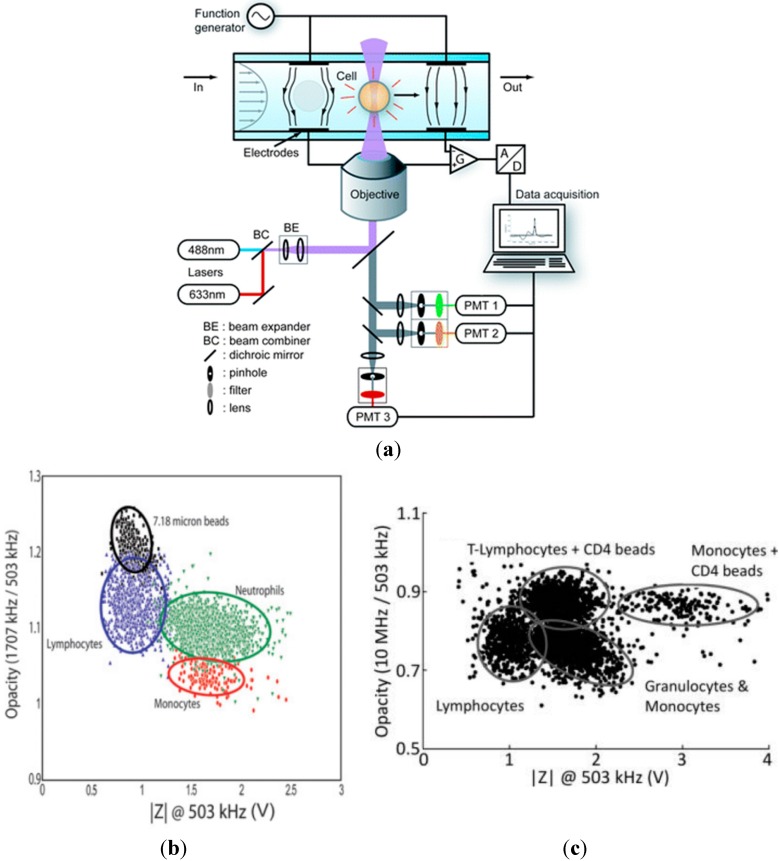
(**a**) The first-generation microfluidic impedance and fluorescent flow cytometry where a cell flows between two pairs of electrodes and the optical detection region composed of dual laser excitation, three color detection and dual frequency impedance measurement; (**b**) Impedance and fluorescent measurement results. Based on low frequency impedance amplitudes, lymphocytes can be differentiated from monocytes and neutrophils due to significantly smaller cell sizes. High frequency impedance amplitudes were used to differentiate neutrophils from monocytes due to significant differences in membrane capacitance. Note that these impedance based classification were validated by the simultaneous fluorescent detection by fluorescently labelling whole blood cells; (**c**) Whole blood cells mixing with CD4 antibody coated beads were characterized by the microfluidic impedance and fluorescent flow cytometry where lymphocytes, lymphocytes + CD4 beads, granulocytes & monocytes and monocytes + CD4 beads were successfully classified and confirmed by simultaneous fluorescent characterization [10,11].

In order to enhance the functionality of microfluidic impedance flow cytometry and provide a more comprehensive understanding on cellular biophysical and biochemical properties, Morgan *et al.* integrated the functions of impedance measurement and fluorescence detection in a microfluidic impedance and fluorescent flow cytometry [8,10,11,35,81,86,87,88,95,105,106,107,108,109,110,111,112,113,114,115,116,117,88,95,105]. Figure 6a shows the first-generation microfluidic platforms capable of characterizing both cellular electrical and optical property consisting of dual laser excitation, three color detection and dual frequency impedance measurement [10]. As the first demonstration, whole blood cells were successfully classified by the microfluidic impedance and fluorescent flow cytometry (see Figure 6b) [10]. In this study, the lymphocytes were differentiated from monocytes and neutrophils due to their significantly smaller cell sizes based on impedance data at 503 kHz. In addition, the neutrophils were differentiated from monocytes due to their significant differences in membrane capacitance based on impedance data at 1.707 MHz.

Furthermore, the whole blood cells mixed with CD4 antibody coated beads were successfully characterized by the microfluidic impedance (frequency: 503 kHz and 10 MHz) and fluorescent flow cytometry. In this work, the lymphocytes, lymphocytes + CD4 beads, granulocytes & monocytes and monocytes + CD4 beads were classified. This method was found useful for CD4^+^ T-lymphocyte counting (see Figure 6c) [11]. Note that these impedance based cell type classification were confirmed by the simultaneous fluorescent measurement.

**Figure 7:**
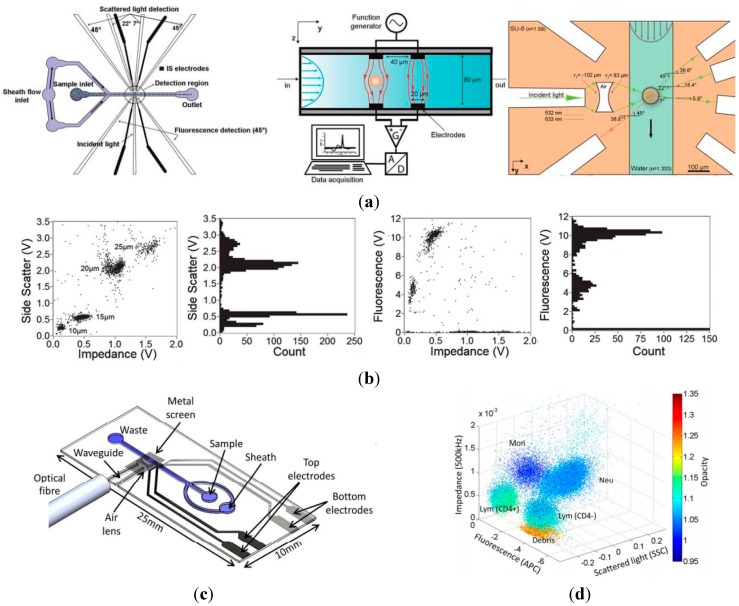
(**a**) The second-generation microfluidic impedance and fluorescent flow cytometry with on-chip optical components where a groove in SU-8 holds a fiber to launch incident light, which is then focused into the channel using an air compound lens. Fibers at various angles are used to collect fluorescence emission, optical extinction signal loss, and side scattered light, respectively; (**b**) Side scattered light, fluorescence and impedance data based classification of a mixture of different beads (10 and 15 μm diameter fluorescent, 20 and 25 μm diameter plain); (**c**) A new microfluidic impedance and fluorescent flow cytometry with on-chip waveguides in a sheath-less manner, which can effectively address misalignment of the optical fibers, incident light scatter from multiple interfaces and signal dependent on particle positions; (**d**) The 3-D scatter plot for CD4 labelled white blood cells based on parameters of side scatter light, fluorescence, and two-frequency impedance data. Both side scattered light and low frequency impedance data provide information on cell sizes, which discriminate smaller lymphocytes from granulocytes. High-frequency impedance data discriminates monocytes from neutrophils due to differences in cell membrane capacitance while CD4 labelled white blood cells were distinguished from white blood cells without CD4 labelling based on fluorescent data [86,87].

In the first-generation microfluidics-based impedance and fluorescent flow cytometry, laser excitation and fluorescent collection was implemented by using optical lens and thus only fluorescent signals can be obtained while other optical parameters such as side scattered light cannot be acquired. In order to address the limitation, Morgan *et al.* proposed the second-generation impedance and fluorescent flow cytometry where optical fibers were integrated into the microfluidic platform enabling the simultaneous measurement of impedance signals (at two frequencies) and optical signals (e.g., side scattered light and fluorescence) [86,87]. Figure 7a shows the microfluidic impedance flow cytometry with on-chip optical components. More specifically, a groove in SU-8 material holds a fiber to launch incident light perpendicular to the channel, which is focused into a sheet across the width of the channel using an air compound lens. Fluorescent emission is then collected with the fibers placed in two grooves on the same side as the incident light (at 135°). A 7° fiber is used to measure the optical extinction signal and the light loss due when a particle passes through an incident beam. Furthermore, two more collection fibers placed at 22.5° and 45° were designed to measure side scattered light [86]. The reported microfluidic platform was used to classify a mixture of beads (the fluorescent beads with 10 and 15 μm in diameter, and the plain beads with 20 and 25 μm in diameter). Figure 7b shows that the beads with four different sizes can be distinguished from optical side scattered light, but the impedance signals provide much better discrimination between the populations. The fluorescence signals from the 10 and 15 μm beads provide easy discrimination in this platform.

In 2014, Spencer and Morgan proposed a novel microfluidics-based impedance and fluorescent flow cytometry capable of measuring four different parameters, namely fluorescence, large angle side scattered light and dual frequency electrical impedance (electrical volume and opacity) (see Figure 7c) [87]. In this study, on-chip waveguides were used to replace the inserted fibers described in the previous study, which can effectively address the issues of optical fiber misalignments and incident light scattering from multiple interfaces. In addition, a sheath-less particle focusing technique was used and thus hydrodynamic focusing is no longer required. Figure 7d shows a 3-D scatter plot for a CD4 labelled white blood cell sample based on parameters of side scattered light, fluorescence, and two-frequency impedance data. Both side scattered light and low frequency impedance data at 0.5 MHz provide information on cell sizes, which separate smaller lymphocytes from granulocytes. High-frequency impedance data at 2 MHz separate monocytes from neutrophils due to differences in cell membrane capacitance while CD4 labelled white blood cells were distinguished from white blood cells without CD4 labelling based on fluorescent data.

## 5. Integrated Point of Care System Based on Microfluidic Impedance Flow Cytometry

Diagnostic testing at or near the site of patient care is often termed as “near-patient” or “point-of-care” (POC) testing, which can be facilitated by microfluidic technologies [118,119,120,121,122,123]. Blood cell counting, as the most common clinical indicator of patient health, is one area where microfluidics based POC systems are expected to bring significant advancements [124,125,126]. Due to the advantages of compactness, low cost and no requirement for optical interfaces, microfluidic impedance flow cytometry has been integrated with sample pretreatment components to enable whole blood cell counting in the POC manner [7,88].

**Figure 8:**
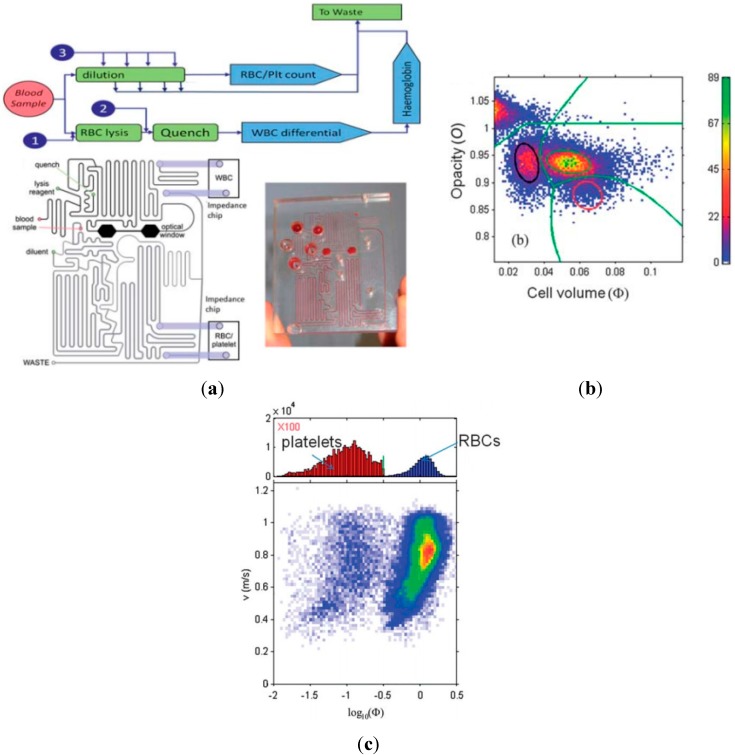
(**a**) The integrated point of care system based on microfluidic impedance flow cytometry enabling whole blood cell counting. The integrated system consists of two parts, an impedance measuring chip and a microfluidic sample preparation block. The bottom arm performs analysis of white blood cells with erythrocytes lysed while the upper arm performs counting of red blood cells and platelets; (**b**) The impedance scatter plot of cell membrane opacity *vs.* the electrical cell volume for classification of three main subpopulations of white blood cells (lymphocytes, monocytes and neutrophils); (**c**) Counting of red blood cells and platelets was performed based on single-frequency impedance data, where the cells are easily differentiated by sizes [88].

In 2011, Morgan *et al.* proposed an integrated microfluidic platform based on impedance flow cytometry, enabling the counting of 3-part differential leukocytes (granulocyte, lymphocyte and monocyte), as well as erythrocytes and platelets from raw blood samples [8,88]. As shown in Figure 8a, the integrated system consists of two parts: an impedance detection chip and a microfluidic sample preparation block. The microfluidic sample preparation block performs whole blood loading, pre-treatment and dilution into two separate fluid channels for impedance characterization, respectively. The bottom arm performs analysis of white blood cells with erythrocytes lysed while the upper arm performs counting of red blood cells and platelets.

Figure 8b shows the impedance scatter plot of cell membrane opacity (the ratio of impedance measured at 1.7 to 0.5 MHz) *vs.* the electrical cell volume (impedance magnitude at 0.5 MHz) for white blood cells. Consistent with previous studies [10,11], the three main subpopulations (lymphocytes, monocytes and neutrophils) are clearly separated while the top left region represents red blood ghost cells and other debris that are not completely eliminated by the on-chip lysis. Counting of red blood cells and platelets was performed using a single frequency of 0.5 MHz, where the cells are easily differentiated by sizes (see Figure 8c). Due to the relatively low number of platelets, platelet concordance was conducted, and the results showed an excellent linearity between the absolute platelet counts obtained from the impedance cytometry system and the hematology analyzer in hospitals.

**Figure 9:**
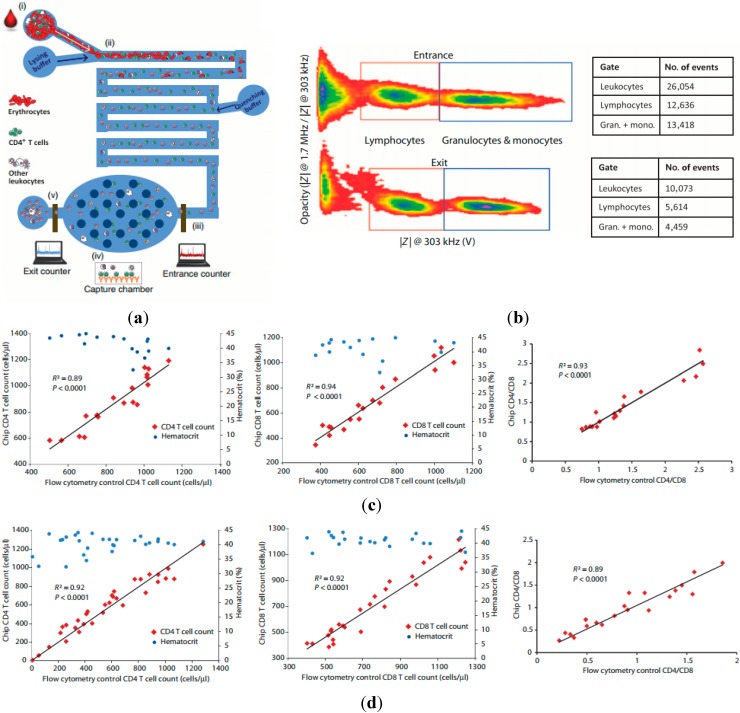
(**a**) The integrated point of care system based on microfluidic impedance flow cytometry enabling CD4^+^ and CD8^+^ T Lymphocyte counting. The integrated microfluidic device relies on five on-chip modules that are, in sequence, chemically lyses erythrocytes, quenches lysis to preserve leukocytes, enumerates cells electrically, depletes the target cells (CD4 or CD8) with antibodies, and enumerates the remaining cells electrically. Target cell depletion was accomplished through shear stress-based immunocapture; (**b**) Scatter plots of opacity *vs.* the low-frequency impedance amplitude for white blood cells before and after CD4 and CD8 depletion; CD4^+^ and CD8^+^ T cell count results between chip and flow cytometry control with a close match using healthy (*n =* 18) (**c**) and HIV-infected patient (*n =* 32) (**d**) blood samples, respectively [7].

In 2013, Bashir *et al.* proposed a microfluidic CD4^+^ and CD8^+^ T Lymphocyte counter for point-of-care HIV diagnostics targeting raw whole blood samples [7,127,128]. As shown in Figure 9a, the integrated microfluidic device is based on differential electrical counting and relies on five on-chip modules that, in sequence, chemically lyses erythrocytes, quenches lysis to preserve leukocytes, enumerates cells electrically, depletes the target cells (CD4 or CD8) with antibodies, and enumerates the remaining cells electrically. Target cell depletion was accomplished through shear stress-based immunocapture, and antibody-coated microposts were used to increase the contact surface areas and enhance the depletion efficiency. Based on the differential electrical counting method, which relies on two-frequency impedance data to classify lymphocytes, monocytes and neutrophils, CD4^+^ and CD8^+^ cell difference before and after the target cell depletion region was quantified (see Figure 9b).

Figure 9c,d show CD4^+^ and CD8^+^ T cell count results between chip and flow cytometry control with a close match using healthy (*n* = 18) and HIV-infected patient (*n =* 32) blood samples, respectively. By providing accurate cell counts in less than 20 min, this approach can be potentially implemented as a handheld, battery-powered instrument that would deliver simple HIV diagnostics to patients anywhere in the world, regardless of geography or socioeconomic status.

## 6. Conclusions and Outlook

In this review, recent developments in the field of microfluidic impedance flow cytometry have been discussed from four perspectives: (1) early developments of microfluidic impedance flow cytometry for single-cell electrical property characterization; (2) microfluidic impedance flow cytometry with enhanced sensitivity; (3) microfluidic impedance and optical flow cytometry for single-cell analysis and (4) integrated point of care system based on microfluidic impedance flow cytometry.

From the aspect of technical development, microfluidic impedance flow cytometry enabling high-throughput characterization of size-independent intrinsic cellular electrical properties (e.g., specific membrane capacitance, a throughput of ~1000 cells per second) should be under intensive research. The majority of reported microfluidic impedance flow cytometry can collect cellular electrical properties in a high-throughput manner, which, however, are only capable of reporting size-dependent electrical properties (e.g., impedance values at several specific frequencies). Although these parameters can indicate membrane capacitance and cytoplasm resistance, they are dependent on cell sizes and specific experimental conditions (e.g., channel geometries and electrode dimensions). Since these parameters do not directly reflect intrinsic cellular electrical properties, it would be difficult to evaluate cellular status and classify cell types based on these parameters.

Recently, impedance spectroscopy and the constriction channel design were combined, enabling the quantification of C_specific membrane_ and σ_cytoplasm_ from hundreds of cells [85,103]. In addition, a microfluidic platform was developed where the cross-sectional area of the constriction channel is under regulation, effectively addressing the issue of constriction channel blockage [104]. However, the throughput of such microfluidic devices is roughly one cell per second, which is still low as compared to the conventional flow cytometry (~1000 cells per second). Thus, further technical development should focus on microfluidic impedance flow cytometry enabling high-throughput size-independent intrinsic electrical property characterization of single cells.

From the perspectives of clinical applications, microfluidic impedance flow cytometry can be used to classify tumor cells, stem cells, and blood cells in a label-free manner. In the field of tumor cell classification [5,64,83], paired high- and low-metastatic carcinoma strains and tumor cells as well as their counterparts with single oncogenes under regulation were successfully classified based on cellular electrical properties [5]. Further studies should be conducted to characterize electrical properties of human tumor samples and evaluate the feasibility of tumor classification based on cellular electrical properties.

As to the stem cell classification, undifferentiated and differentiated mouse embryonic carcinoma cells (P19) based on impedance data at 50 kHz, 250 kHz, 500 kHz and 1 MHz were differentiated [6] where it was speculated that the capacitance of stem cells can vary as they experience various stages of differentiation. These results provide some preliminary data along this direction but more data are needed for a decisive conclusion. For example, during stem cell differentiation, impedance data should be collected at multiple time points. This can help sketch the trend in how electrical properties of stem cells evolve as they differentiate into adult cells. Furthermore, electrical properties of human rather than mouse stem cells should be characterized to further evaluate the possibility of stem cell classification based on cellular electrical properties.

In the field of red blood cell classification based on cellular electrical properties [9,84], in 2013, Chandrakasan *et al.* developed a microfluidic impedance flow cytometry capable of differentiating *P. falciparum* infected red blood cells from uninfected red blood cells based on amplitude and phase data at 2 MHz. However, multiple-frequency impedance data are suggested to further evaluate the electrical properties of various types of red blood cells. For white blood cell differentiation, since it has a close relationship with point of care applications, intensive research efforts have been devoted [7,8,10,11,88] (e.g., CD4^+^ T lymphocyte counting [7]). Further studies should compare these approaches with other point of care methods and test a large number of human samples with statistical significance.
